# Veterinary Legislation in Illinois

**Published:** 1899-06

**Authors:** 


### VETERINARY LEGISLATION IN ILLINOIS.

MUCH comment of varying character has been indulged in relative to the recent legislation in the "Prairie" State establishing a State Board of Veterinary Medical Examiners.

The feature of the law making the board subject to the control and direction of the State Livestock Board of Commissioners has been severely criticised, and, from the stand-point of those of the profession who believe that the latter should stand or fall on its own merits, perhaps, rightfully criticised; but those more experienced in legislative battles realize that such a position, at times, means repeated and almost unending failures, the latter far more discouraging to the profession than the hopes of any legislation, even ideal in character, to correct the evils that legislation is primarily intended to relieve. Those who have fought the legislative battles, National and State, have learned the one hard fact that the entering wedge of legislation on any new lines is the most difficult to obtain, and only gained after the hardest and most conscientious work. Its after-amendment and correction are readily yielded to, compared with the difficulties encountered at every step of new legislation. In these modern times the pitfalls of any proposed legislative measure are so numerous that only those who have directed these battles and tented or lived on the legislative field can foresee them.

The peculiar condition existing in Illinois, after a period of dangerous legislation indulged in by a prior Legislature, followed by a general upheaval all along the line of those responsible for previous legislative measures, abortive and dangerous in character to the welfare of the people, made any new legislation almost impossible of attainment. The generally satisfactory record of the Livestock Board of Commissioners seemed to be the only open way for legislation of this character; hence the decision of the profession as expressed by Association consideration. While many will still feel that one should not "play second fiddle," still every appreciation should be shown to those who labored so earnestly and long for the obtaining of this recognition, and final judgment as to its wisdom and value should be withheld until those who have so ardently pinned their faith to this measure have had an opportunity of testing its value by its execution. Much will depend upon how faithfully the profession of the State sustain and uphold the board and the character of support they give the purposes of the measure.

